# Endpoint Detection Based on Optical Method in Chemical Mechanical Polishing

**DOI:** 10.3390/mi14112053

**Published:** 2023-11-02

**Authors:** Fangxin Tian, Tongqing Wang, Xinchun Lu

**Affiliations:** State Key Laboratory of Tribology, Tsinghua University, Beijing 100084, China

**Keywords:** chemical mechanical polishing, endpoint detection, optical trace

## Abstract

Endpoint detection is an important technology in chemical mechanical polishing (CMP), which is used to capture the material interface and compensate the variations of consumables and incoming wafer thickness. This paper aimed to apply optical detection in metal CMP. An in situ optical measurement system was developed for a 12-inch CMP tool. Kinematic analysis of the scanning trajectory of the laser device indicated the relative position relationship between the device and the wafer. Average optical data within the wafer described the material removal of metal CMP. Furthermore, optical data and location described the non-uniformity of the entire wafer surface. In this research, the polishing condition and the residual of the wafer edge are characterized by optical trace. Pauta Criterion is used to discriminate the inflexion point of the material interface. The results reveal that the interface capture is accurate and effective.

## 1. Introduction

Chemical mechanical polishing (CMP), as a material processing technology, mainly removes materials and residues from the device surface through mechanical force and chemical reactions [1]. It can obtain a planar, smooth, and damage-free wafer surface [2,3]. Endpoint detection (EPD) is one of the most important challenges for CMP. In the field of chemical mechanical polishing, endpoint detection is a crucial step [4]. It focuses on identifying whether the polishing process has reached the desired endpoint, that is, that the surface uniformity, profile, and material removal have all met the expected requirements, thus improving wafer yield and product consistency [5].

However, traditional endpoint detection methods have certain limitations, mainly in the following aspects. Firstly, traditional endpoint detection methods mainly rely on the experience and observation of the operators, which subjectively can lead to significant errors in the judgment of the polishing endpoint. Secondly, traditional endpoint detection methods, such as frictional force [6], pad temperature [7], acoustic emissions [4,8,9], and Cu-ion concentration [10,11], cannot monitor the polishing process and internal changes in real-time, and can only judge the endpoint based on the surface state after polishing, which is not accurate and reliable for some special and complex materials. In addition, traditional methods cannot evaluate and monitor the uniformity of material removal and surface profile effectively.

In order to overcome these limitations, optical methods are widely used in polishing endpoint detection [12,13]. Optical methods have advantages such as non-contact, high resolution, and real-time, which can provide more accurate and reliable endpoint detection results [14]. Optical sensors can obtain information about the material by the characteristics of light reflection, scattering, and transmission [12]. These characteristic parameters can monitor the polishing process and surface condition. In recent years, optical methods have made progress in the field of polishing endpoint detection. However, there are still challenges and problems that need further research.

Moreover, the eddy current method is also widely used in the industry. In recent years, eddy current technology has been used to measure the thickness of metal film [15,16]. It has many advantages such as low cost, high sensitivity, a large measurement range, and insensitivity to contaminants. It is also a non-contact measurement and very suitable for semiconductor metal measurement. However, for the measurement of extremely thin metal layer or the identification of metal interface layer, it is easy to be affected by slurry, temperature, environment, and other factors, and the accuracy of interface identification is not as good as that of the optical method.

This study aims to research optical endpoint detection technology. We first introduce the purpose of endpoint detection, analyze the principle of optical detection, and describe in detail how to use the principle of light reflection to identify the change in metal materials. Based on this, an optical endpoint detection system is developed and integrated on a 12-inch CMP device. We discuss the design of the optical module and the selection of key components, and then use this set of systems for experiments, analyze the original data, and fit the optical real-time morphology. Finally, the method of interface recognition by morphology is discussed.

## 2. Measuring Principle

Optical metrology is based on two optical principles, reflection of an optical beam from a metal layer and optical interference during reflection of the beam from a transparent, beam-splitting layer. This article focuses on metal material measurement. The main application of optical detection based on reflection principle is to detect the metal polishing in real time. It can monitor the polishing status accurately and tell the CMP control system when to stop polishing. Process control with endpoint detection instead of a fixed polishing time can avoid the over polishing and under polishing effectively.

The most significant optical properties of metals are high reflection, strong absorption, and transparency when the film thickness is less than 30 to 40 nanometers. These properties are related to the good conductivity of metals. Materials with higher conductivity have limited penetration depth and higher reflectivity. Therefore, most reflective metal films use materials such as silver (Ag), copper (Cu), gold (Au), and aluminum (Al) with high conductivity. Table 1 shows the reflectivity of common polishing materials in the CMP metal process at a wavelength of 650 nm, referring to the optical manual [17,18] and the parameters and formulas related to the optical constants of thin film materials. It should be noted that these reflectance values are approximate, and the actual values may be affected by factors such as material quality, surface processing technology, lighting conditions, etc.

**Figure 1:**
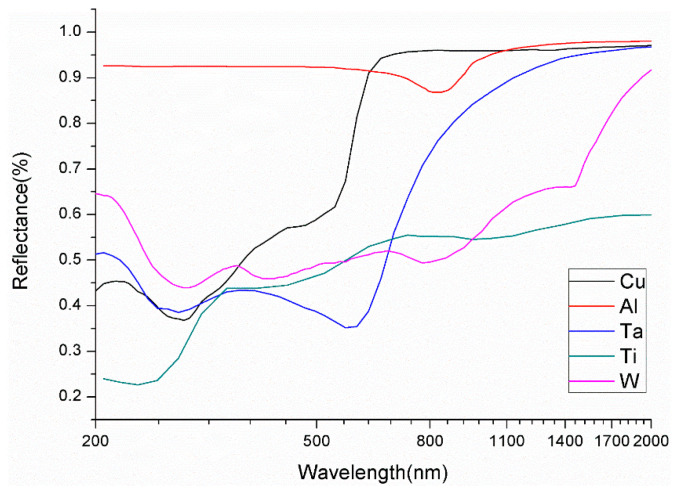
Reflection spectra of metal materials at 300 K.

The reflectivity of a material is closely related to its composition and structure and is also affected by temperature and wavelength. Figure 1 shows the reflection spectrum of a metal material at 300 degrees Kelvin. In the figure, it can be seen that copper, aluminum, and other metal materials have higher reflection spectral characteristics within the wavelength range of visible red light (usually 600 nm to 700 nm). Meanwhile, the reflectivity of barrier materials such as tantalum/tantalum nitride (Ta/TaN) and titanium/titanium nitride (Ti/TiN) is relatively low. This feature is applied in metal endpoint detection.

**Figure 2:**
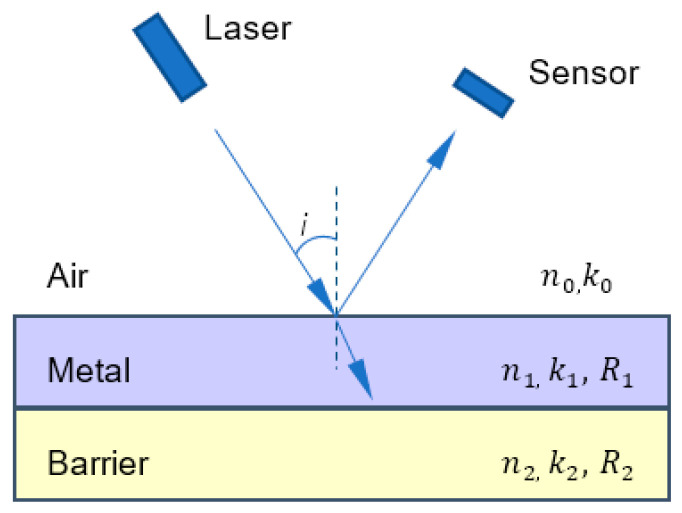
Reflection from multi-layer of wafer.

Deposit thin films such as a barrier layer and metal layer on a silicon substrate, as shown in Figure 2. Each layer of thin film has its own optical constants *n*, *k*, and reflectivity *R*. As incident light, the laser beam scans the metal surface, the reflected light is received by a photoelectric sensor, reflection intensity $I_{r}$ can be defined as:

(1) $$I_{r}=I_{i}R$$

where $I_{i}$ is the intensity of the incident light, *R* is the reflectivity of the film, and $R_{1}>R_{2}$. The reflected light intensity only depends on the reflectivity when the incident light intensity is constant. During the polishing, the removal material changes from the metal layer to the barrier layer. Due to the fact that the reflectance of the barrier layer is much lower than that of the metal, the photoelectric sensor will detect a sharp decrease in the intensity of the reflected light, which is the endpoint of the metal layer removal.

## 3. Design of Optical Detection System

**Figure 3:**
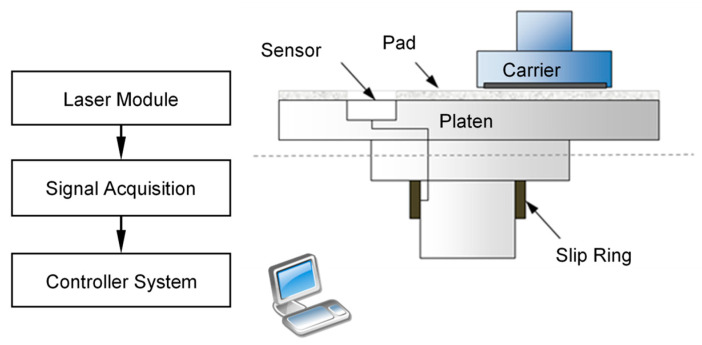
Optical detection system on CMP tool.

An optical detection system on a 12-inch CMP tool is designed as shown in Figure 3. This system includes a laser module, a signal acquisition unit, and a system controller. The laser module is embedded in the platen which is covered by a window pad. Under the components of platen, a slip ring is installed for the optical signal transmission. The collected voltage signal is processed and sent to the system controller to make optical profiles.

The design optimization of optical modules and the selection of components are very important for optical detection systems. Appropriate light sources and photoelectric sensors need to be selected to ensure detection accuracy and resolution. In addition, it is necessary to optimize the distance and angle range of light source detection to adapt to different device platforms. Meanwhile, calibration is also needed to ensure measurement consistency. 

### 3.1. Optical Module Design

**Figure 4:**
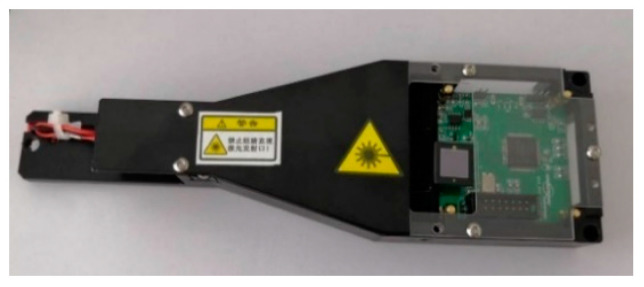
Optical module.

The optical module is the most important component, as shown in Figure 4. The emission of light sources, detection of reflected light, and conversion of photoelectric signals are all completed in the optical module. The optical module is composed of main components such as lasers, planar mirrors, photoelectric sensors, and circuit boards. The light source should be a monochromatic laser with good directionality, monochromaticity, and coherence. Compared with other light sources, monochromatic laser sources have a narrower light source bandwidth and concentrated light intensity, which are conducive to the detection of film thickness.

**Figure 5:**
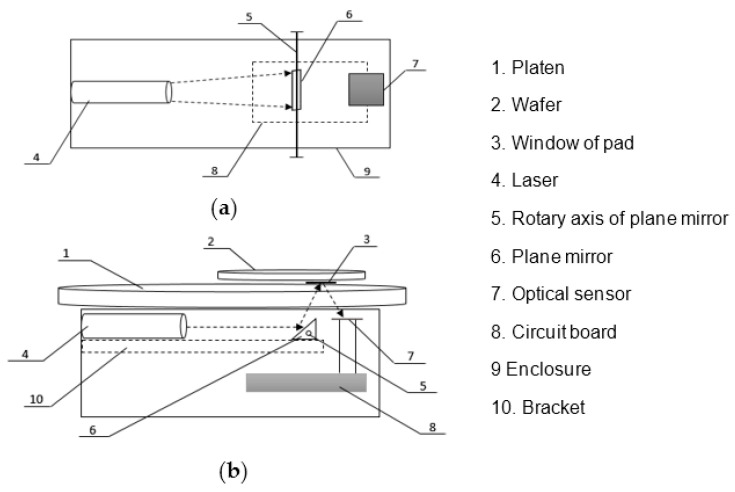
Schematic diagram of optical module: (**a**) top view; (**b**) front view.

Incident light of the laser is reflected by a plane mirror and illuminates the wafer surface through the window of the polishing pad. Reflected light is then projected onto the photodiode through the pad window so as to detect the intensity of the reflected laser beam. The width of the pad window is very narrow, usually about 10 mm, which needs high requirements for the angle of the incident beam. The incident angle must be accurate and constant to make sure that incident and reflected light path could pass through the window without being blocked. Therefore, we consider a double-layer design to reduce device size. As shown in Figure 5, the circuit board is placed in the lower layer, and the photoelectric sensor is led out to the upper layer, while the laser and plane mirror are placed on the middle partition.

This layout makes it possible to install the device along the radial direction of the platen. In this case, the laser line is incident from the short side of the window, so that the obstruction of the reflected light could be avoided completely. Generally, the photosensitive area of optical acquisition sensor is 10 mm × 10 mm, so the length of the laser beam should be greater than 10 mm, and it should not be too long, which will cause a large loss of optical energy. Here we use a custom-made 650 nm red laser of line laser to produce an optical beam in front of the laser. In this way, it is no longer necessary to adjust light beams with extra lens assembly, which simplifies the optical path. By adding a rotation axis to the plane mirror, the incident laser angle could be adjustable and not be affected by the distance between the pad window and plane mirror. It brings high fault tolerance and adjustability and makes the module suitable for different CMP platforms.

### 3.2. Selection of Optical Device

The following section will introduce the parameters and selection of lasers and photoelectric sensors in the measurement module.

The light source of the detection system needs to include the following conditions: good collimation of the beam, stable power, and wavelength should be within the visible wavelength range. From the previous section, it can be seen that a red laser with a monochromatic wavelength greater than 600 nm should be selected as the light source in order to achieve metal interface layer detection.

**Figure 6:**
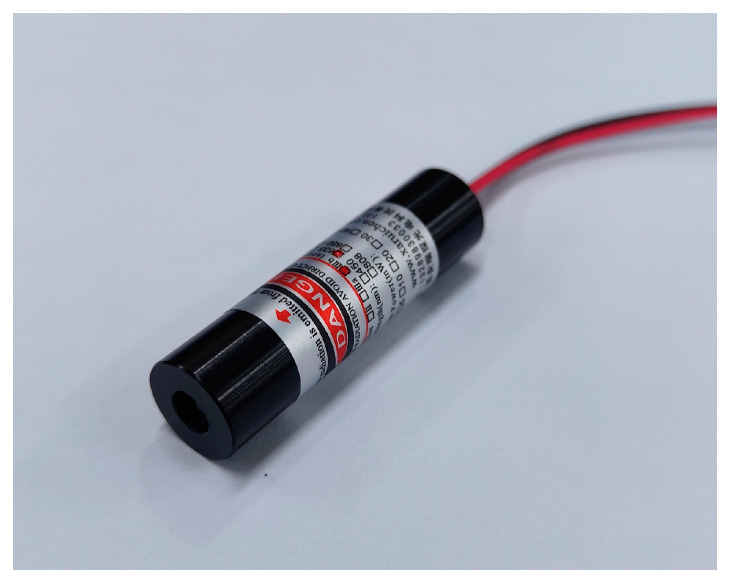
Optical laser.

The laser of the system in this paper uses a straight-line laser with a red light, as shown in Figure 6. The optical module uses laser diode, high-performance automatic power control (APC), or automatic current control (ACC) drive circuits and optical coated glass groups. These optical modules have characteristics such as high stability, good consistency, strong anti-interference, and long service life. The laser uses tubes with different powers, collimating lenses with different parameters, and cylindrical mirrors to produce a uniform straight-line laser. The beam curvature at a working distance of 5 m is not more than 1 mm. The power is 3 mv, which meets the safety standards of IIIA. The output wavelength of this low-power semiconductor laser is also relatively stable, meeting the measurement requirements of the system. The specifications are shown in Table 2.

Photoelectric sensors are devices used in systems to receive optical signals, using photosensitive elements to receive optical signals and convert them into electrical signals. Optical detection usually uses photodiodes as photosensitive components. In this article, silicon photodiodes are selected, and their current is proportional to the intensity of light. They are universal photometers suitable for laser intensity detection, with low noise, a low dark current, and high moisture resistance. They have high sensitivity in the visible light band, and specific parameters are shown in Table 3.

### 3.3. Calibration with Fixtures

**Figure 7:**
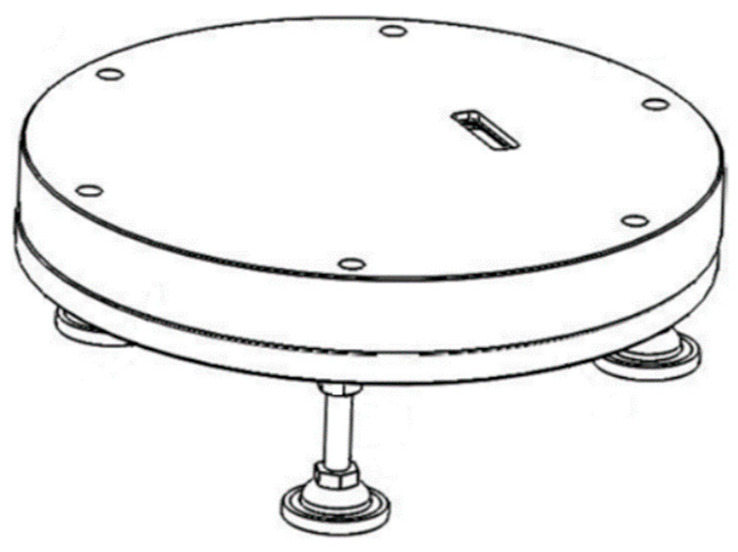
Calibration fixtures.

The installation of the optical detection module requires confirmation of the incident angle. Design a fixture as shown in Figure 7, which can simulate the actual installation position and optical path of the module inside the platen. The fixture consists of three parts: support legs, tray, and upper platen. The module is placed on the fixture tray, and a chamber is designed in the upper platen to accommodate the optical module. The chamber size matches the optical module. The tray and upper platen are positioned through a pin hole structure and locked with the support legs.

There is a through-hole in the platen that can make a transparent area. The O-ring compresses and seals the frosted glass in the transparent area, while also providing waterproofing. It can not only make the incident light pass through the transparent area and incident on the material surface, but also reduce the actual output power of the light source through scattering and absorption.

## 4. Data Acquisition and Signal Processing

### 4.1. Scanning Trajectory Analysis

**Figure 8:**
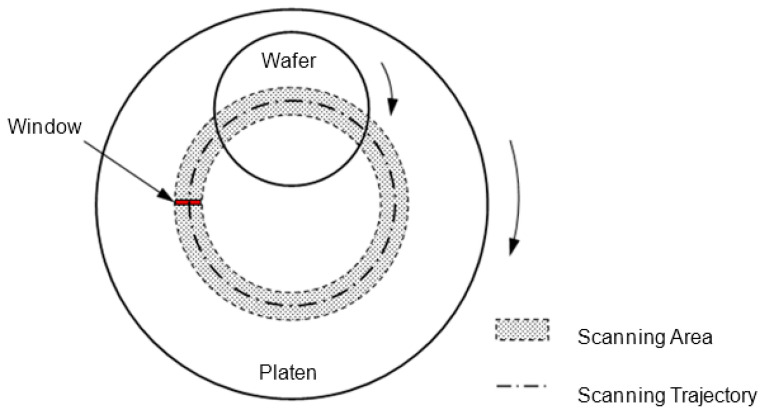
Scanning area and trajectory of laser window.

During the CMP process, the platen and the wafer rotate in the same direction, while the wafer carrier reciprocates along the radial direction of the platen [19]. The laser window rotates with the platen to scan across the wafer surface and we will obtain an arc-shaped scan line. When the wafer rotates, the arc-shaped scan lines will be evenly distributed on the whole surface of the wafer, so as to obtain the overall morphology of the wafer. Since the optical signal is the result of the combined action of the whole laser beam, the center point of the laser window is defined as the action point of the laser beam, and the scanning trajectory can be obtained as shown in Figure 8.

**Figure 9:**
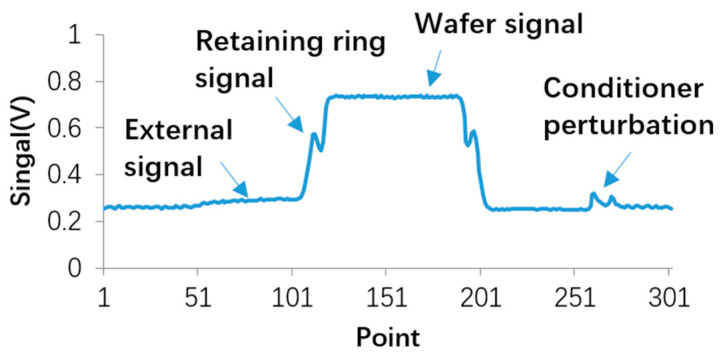
Optical signal curve during one revolution of platen.

The raw data of the optical signals are collected continuously to generate scanning trajectories. Figure 9 shows the optical signals during one revolution of the platen, including the wafer signal, retaining ring signal, and external signal.

The wafer signal is at a high level, and the intensity of the signal depends on the material of the wafer metal layer. The retaining ring signal lies just around the shoulder of the curve shown in Figure 9. The external signal is at a low level, mainly affected by sunlight scattering. The perturbation on the right side of the external signal is caused by the metal material of pad conditioner.

Usually, automatic calibration is used to normalize optical signals and convert voltage signals into dimensionless parameters. We use a metal wafer (with a film thickness greater than 1000 Å to ensure its opacity) to remove all the surface metal layers until the barrier layer is exposed, and then select the optical signals on the metal layer and barrier layer for calibration.

### 4.2. Profile Fitting

In order to obtain a real-time optical profile, it is necessary to do the relative position analysis on the sensors. The optical scanning area and scanning trajectory have been given in Section 4.1., and this section discusses the relative position relationship between the points on the trajectory and the wafer.

Assume point P as a point on the scanning trajectory with a radius *R* from the center of the platen in Figure 8. To obtain accurate sensor position and angle, we use bipolar Hall sensors or ordinary photoelectric switches to calibrate the zero position of the polishing platen. When the center of the optical sensor is located below the wafer and in the radial motion direction of the polishing head, the position angle is set to zero, and the encoder value of the platen motor is marked at this time. During the polishing process, it is necessary to convert the angle *θ* of optical sensors based on the motor encoder value. Define *r* as the relative distance between P and the center position of the polishing head. According to the cosine theorem, it can be concluded that:

(2) $$r=\sqrt{e^{2}+R^{2}+2eR\cos\theta }$$

where *e* is the center distance between the head and platen.

## 5. Results and Discussion

### 5.1. Interface Detection

**Figure 10:**
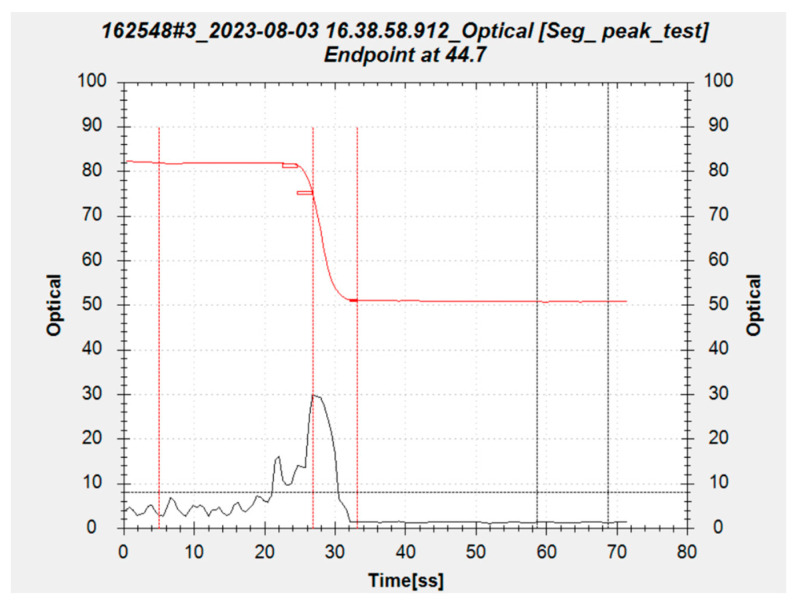
Optical detection curve during polishing process.

For the manufacturing process of 300 mm integrated circuits, the main application of optical endpoint detection is to identify the polishing interface of metal interconnection layers. By identifying the inflection point of the light intensity curve on the wafer, the time when the metal layer is completely removed and the barrier layer is exposed can be accurately controlled. The purpose is to achieve accurate control of polishing time and polishing process. In the polishing process, the wafer signal profile can be captured for each revolution. As shown in Figure 10, the red curve is generated by the mean value of the optical signal profile, it is defined as the detection curve and can be used to characterize metal thickness. The black curve is generated by the max–min difference value of the optical signal profile, the max–min difference value is also called the peak value, it can be used to characterize the uniformity of metal material distribution. The higher the peak value at the end of polishing means the more serious the metal residue. According to the process experience, setting a threshold value for alarm monitoring can effectively monitor the metal residue at the end of polishing.

**Figure 11:**
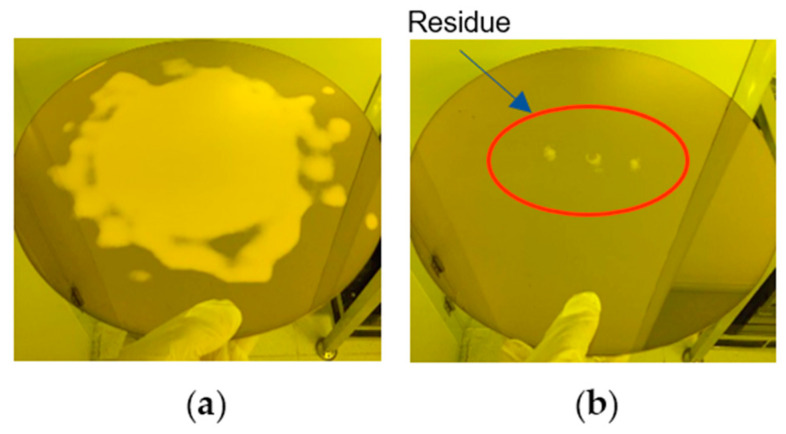
Changes of metal residue during polishing process. (**a**) Metal residue at the first inflection point.; (**b**) Metal residue at the second inflection point.

Taking the low downforce polishing of the copper process as an example, in the initial stage of polishing, there may be an oxide layer on the surface, which affects the optical signal. Therefore, the optical signal in the initial stage cannot be used for identifying inflection points. After surface polishing, it enters a stable removal stage. The reserved copper layer thickness during the low downforce polishing stage is usually between 1000 Å and 2000 Å, and the optical signal during this stage is very stable. Keep polishing until some of the copper film has been removed and the barrier layer begins to appear. The optical signal rapidly decreases, and the detection curve shows the first inflection point. At this time, the metal residue on the wafer is shown in Figure 11a. As the polishing process continues, the proportion of barrier layer materials on the wafer surface gradually increases. When the majority of the copper film is completely removed, the surface material tends to remain unchanged, the optical signal flattens again, and the detection curve shows a second inflection point. At this time, the residual metal on the wafer is shown in Figure 11b. After obtaining the second inflection point, a period of over-polishing time is given to make sure the complete removal of the metal, and the thickness, defects, and electrical parameters of the polished wafer will be well controlled [20]. Traditionally, monitor wafers are used to test the wafer removal rate and evaluate the polishing time required to obtain the target thickness. Due to variations of monitor wafers and removal rates, traditional methods cannot always achieve stable product performance. The optical detection method obtains accurate thickness at the thin barrier layer and minimizes the dishing of the copper structures.

### 5.2. Inflection Point Discrimination

The identification of inflection points can be based on the PauTa criterion. Assuming that the detection data only contain random errors, the standard deviation is obtained by processing the data sample, and then the error range is determined based on the error distribution. Values out of this range are defined as exception values. This discrimination method is only applicable to sample data that conforms to a normal distribution. The sample size needs to be large enough (*n* > 10).

Prepare a copper wafer with a film thickness of 1000 Å, and do the polishing test with deionized water instead of slurry, the downforce can be set to a small pressure of 0.5 psi, the polishing time is 30 s. Obtain the optical measurement signal on the wafer during polishing, record it as a data set $X=\left(x_{1},x_{2},\ldots x_{n}\right)$, and calculate the arithmetic mean value *μ* and overall standard deviation σ of the data set:

(3) $$\mu =\frac{1}{n}\sum_{i=1}^{n}x_{i}$$

(4) $$\sigma =\sqrt{\frac{\sum_{i=1}^{n}{\left(x_{i}-\mu \right)}^{2}}{n}}$$

Review all members in the data set, there is 76.67% of the data in the dataset, whose values are distributed in the range of (*μ* − *σ*, *μ* + *σ*), 93.61% of the data, whose values are distributed in the range of (*μ* − 2*σ*, *μ* + 2*σ*), 99.57% of the data, whose values are distributed in the range of (*μ* − 3*σ*, *μ* + 3*σ*). This means that the optical signal of water polishing is approximating normal distribution, it can be considered that the signal error only contains random error when the polishing material is unchanged, it is also considered that when the signal error is continuously greater than 3*σ*, the polishing material begins to change. According to this criterion, the endpoint detection time can be determined.

### 5.3. Profile Monitoring

**Figure 12:**
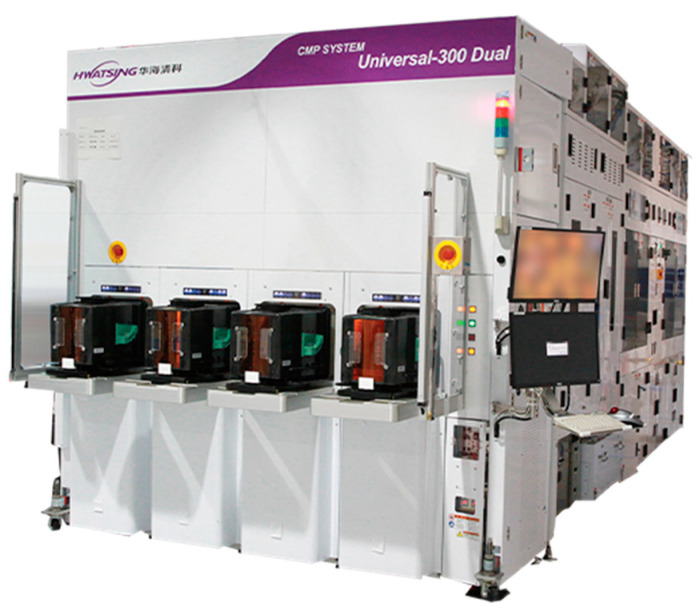
Rotating CMP equipment.

In this study, we use a rotating CMP tool (Hwatsing Technology, Universal-300 Dual), a four-probe metal measuring device (CDE, ResMap 273). The polishing material is two pieces of wafer with copper film. Equipment image is shown in Figure 12.

**Figure 13:**
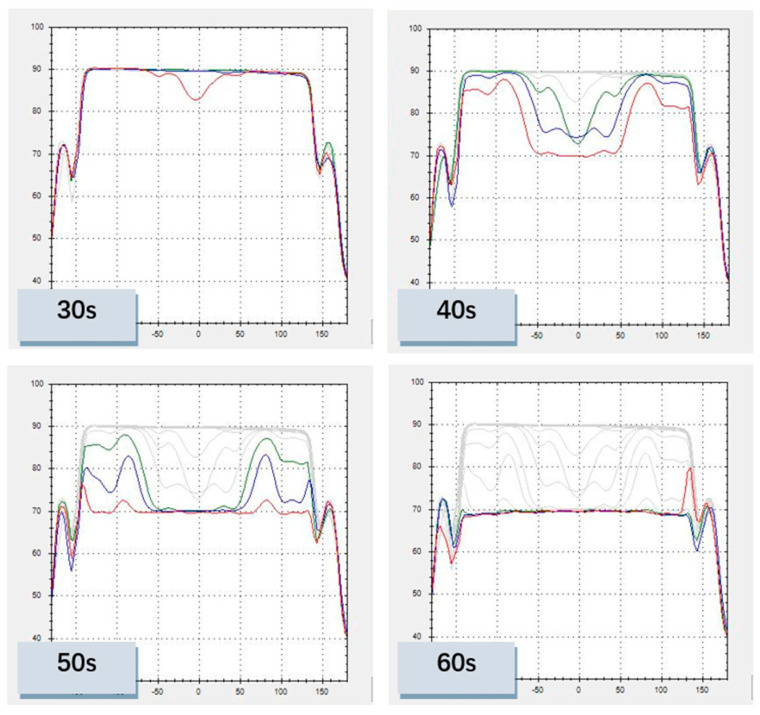
Changes of optical profile during polishing process.

The experiment is performed under the low-down force of 1.5 psi, while the rotation speed of the platen and head are 93 rpm and 87 rpm. The polishing pad IC1000 has been used. Figure 13 shows the variation of the optical profile in this experiment. It reflects the real-time removal of the copper layer. The perturbation of the final profile indicates the residue of the copper film. Process parameters of the experiment are summarized in Table 4.

The scanning profile reflects the non-uniformity of the layer that results from the instability of deposition processes and thickness variations produced by the CMP process. By this optical detection system, an important basis for process optimization is provided.

## 6. Conclusions

This paper analyzed the detection principle of optical endpoint detection. In the red light band, there is a large difference in reflectivity between the metal material and the barrier material. The change of the polishing material can be identified by the variation of the reflectivity. Based on this principle, a 12-inch CMP optical endpoint detection system was developed. Optical signal detection was realized by a 650 nm red laser and photoelectric sensor. Through the analysis and calculation of the relative position of the polishing head and platen, the scanning trajectory of the optical signal is given, and the optical profile fitting is realized. With the real-time optical profile, the endpoint detection curve and peak curve are generated. The peak curve detects the uniformity of the morphology in polishing and alerts the uneven morphology. The optical curve has obvious inflection points, which can identify the metal material layer and barrier layer clearly. This makes it possible to capture the interface of polishing materials accurately. The inflection point capture adopts the PauTa criterion, which defines the moment when the data continuously exceed the normal distribution as the moment when the material begins to change. Real-time morphology monitoring reflects the real-time removal rate, while also providing alarms for residual metal on the wafer. These monitoring functions are particularly important when CMP consumables enter the end of their lifetime.

## Figures and Tables

**Table 1:** Reflectance of common materials at 650 nm.

Material	Reflectivity
Cu	0.943
Al	0.912
Ti	0.538
W	0.518
Ta	0.460

**Table 2:** Parameters of laser.

Parameter	Specifications
Wavelength	650 nm
Safety class Curvature Working voltage	Class II ≤1 mm @ 5 m DC 5 V

**Table 3:** Parameters of photoelectric sensor.

Parameter	Specifications
Window material	Epoxy resin
Photosensitive area Spectral response range Operation temperature	10 mm × 10 mm 340 nm 1100 nm −20 °C–60 °C

**Table 4:** Process parameters of experiment.

Parameter	Specifications
Head Rotation	81 rpm
Platen Rotation	90 rpm
Retaining Ring	3.95 psi
Z1 Down Force	1.73 psi
Z2 Down Force	1.65 psi
Z3 Down Force	1.49 psi
Z4 Down Force	1.39 psi
Z5 Down Force	1.31 psi
Z6 Down Force	1.26 psi
Z7 Down Force	1.23 psi
Slurry	280 mL/min

## Data Availability

The data supporting the reported results by the authors can be sent by e-mail.
